# Synthesis, Experimental and Density Functional Theory (DFT) Studies on Solubility of Camptothecin Derivatives

**DOI:** 10.3390/molecules23123170

**Published:** 2018-12-01

**Authors:** Chin-Hung Lai, Chia-Chin Chang, Yi-Lin Weng, Ta-Hsien Chuang

**Affiliations:** 1 Department of Medical Applied Chemistry, Chung Shan Medical University, Taichung 40201, Taiwan; 2 Department of Medical Education, Chung Shan Medical University Hospital, Taichung 40201, Taiwan; 3 Master Program for Pharmaceutical Manufacture, China Medical University, Taichung 40402, Taiwan; b2625367@gmail.com (C.-C.C.); elaine.850502@gmail.com (Y.-L.W.); 4 School of Pharmacy, China Medical University, Taichung 40402, Taiwan

**Keywords:** acid-catalyzed, condensation, camptothecin, solubility, DFT

## Abstract

Two camptothecin derivatives, 10-cyclohexyl-7-methyl-20(*S*)-camptothecin and 7-methyl-10-morpholino-20(*S*)-camptothecin, were synthesized and their differences in solubility were investigated using four chosen solvent systems. Based on our results, 10-cyclohexyl-7-methyl-20(*S*)-camptothecin exhibited higher solubilities than 7-methyl-10-morpholino-20(*S*)-camptothecin in polar aprotic solvents. However, these two camptothecin derivatives did not exhibit apparent differences in solubility between 5% dimethyl sulfoxide (DMSO)/95% normal saline co-solvent system and 5% dimethylacetamide (DMAC)/95% normal saline co-solvent system. To rationalize their differences in solubility, we also tried to perform a DFT-B3LYP study to investigate their interaction with one water molecule.

## 1. Introduction

**Figure 1:**
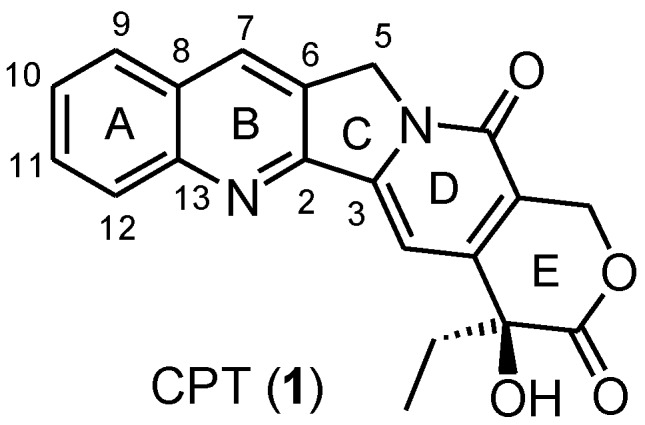
Structure of CPT, **1**.

**Figure 2:**
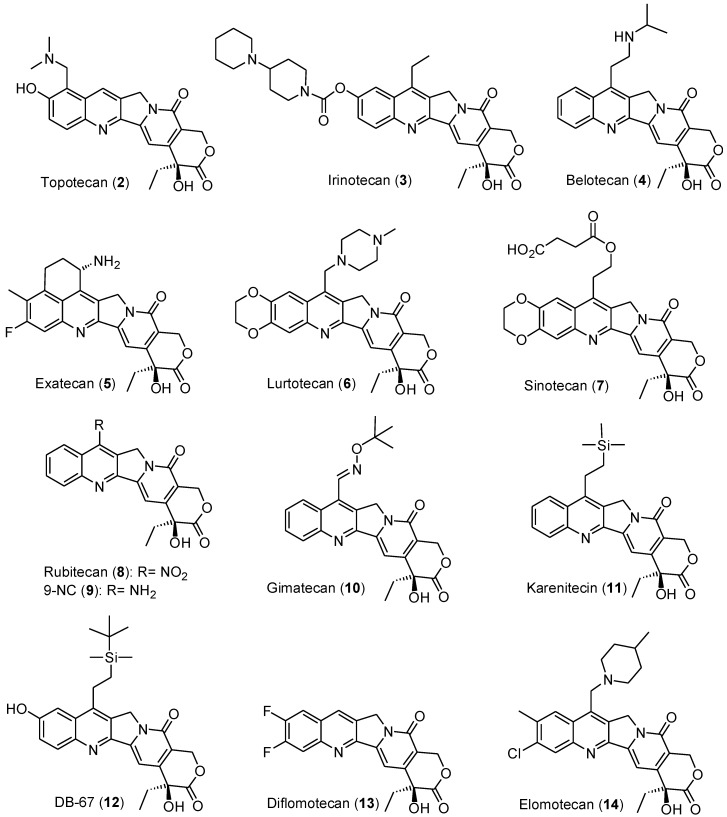
Structures of CPT analogues in use in clinical practice or clinical trials.

Camptothecin (CPT, **1**, Figure 1) is a pentacyclic alkaloid first isolated from *Camptotheca acuminata* by Wall and coworkers in the early 1960s [1]. In the late 1980s, DNA topoisomerase I (topo I) was found to be the therapeutic target for CPT, putting it on the frontline of anticancer drug development [2,3,4,5]. Therefore, its total synthesis, mechanism of action, structure-activity relationship (SAR), and pharmacology have been studied extensively, and preclinical studies and clinical trials of CPT have been carried out. As a result of these research efforts, three CPT analogs, topotecan (TPT, **2**), irinotecan (CPT-11, **3**), and belotecan (CKD-602, **4**) have been used for the clinical treatment of ovarian, small-cell lung, and refractory colorectal cancers [6,7,8]. Their clinical success and intriguing mechanism of action have stimulated great interest in further exploration of CPT derivatives with better antitumor activity; several such derivatives are now undergoing preclinical evaluation. With the three successful compounds **2**–**4** in clinical practice and 10 additional compounds **5**–**14** in clinical trials (Figure 2), CPT analogs have become highly relevant clinical anticancer compounds. Moreover, some excellent reviews on CPT derivatives have been published [9,10,11,12,13,14,15,16,17,18,19,20].

**Figure 3:**
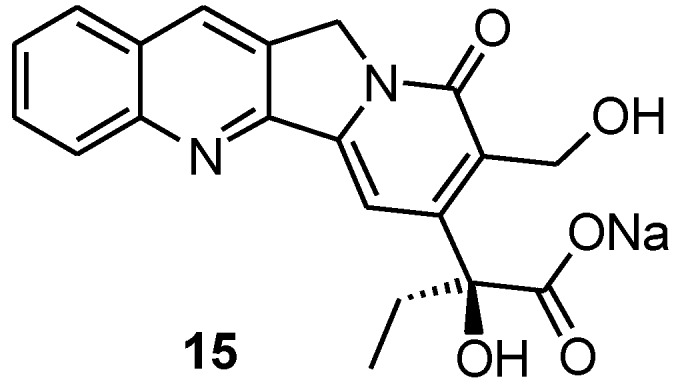
The sodium salt of CPT **15**.

Despite its impressive activity against various cancers in experimental systems, due to its negligible water solubility, CPT was not recognized as a frontline anticancer drug until its mechanism of action was elucidated in the 1980s. In the early 1970s, only the water-soluble sodium salt of CPT (**15**, Figure 3) had been tested in a clinical trial [21]. However, **15** has low efficacy and causes unpredictable and severe side effects, including hemorrhagic cystitis and myelotoxicity. Therefore, clinical trials of CPT were discontinued for more than a decade [22,23]. Thus, the water solubility of CPT has had a great impact on its clinical application. 

**Figure 4:**
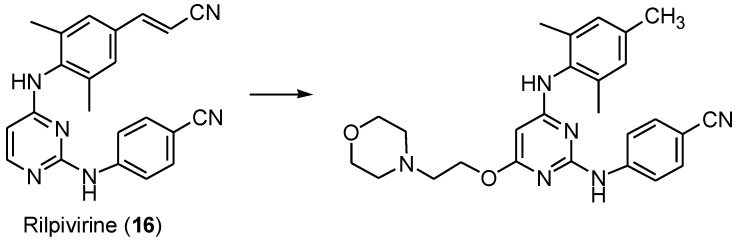
Functionalization of rilpivirine (**16**) with a polar morpholine moiety to improve its water solubility.

Water solubility is a virtual physical and chemical property of organic small molecule drugs and a very important issue in drug design. Drugs with better water solubility often exhibit higher potency and more favorable pharmacokinetic profiles than drugs with lower water solubility. Structural modification is a straightforward and effective method to enhance water solubility. One method of structural modification is to introduce a polar group. The introduction of polar groups can increase the hydration of the compounds and promote the thermodynamic process of dissolution. For example, the introduction of a morpholine ring at the 6 position of the nitrogen-containing parent ring of rilpivirine (**16**, Figure 4) increased its water solubility from only 0.02 μg/mL to 27.3 μg/mL [24]. Rilpivirine is a non-nucleoside reverse transcriptase inhibitor that has good activity against wild-type HIV-1 and two major mutants. The introduction of the polar morpholine ring increased the EC_50_ of rilpivirine from 0.00067 to 0.0086 μmol/L for wild-type HIV-1. 

**Figure 5:**
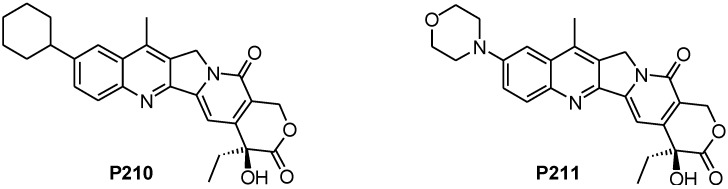
The CPT analogues discussed in this study.

As shown in Figure 2, CPT analogues have been substituted at the 7, 9, 10, or 11 positions. Numerous SAR studies have shown that 7-, 9-, and 10-substituted CPT analogs are better tolerated or have higher anticancer activity than the parent compound, CPT. For example, the hydrolysis product and active metabolite of irinotecin (**3**), 7-ethyl-10-hydroxycamptothecin, in which the carbamate group has been removed and an OH group is present at position 10, could possibly be important in terms of either increasing the water solubility or decreasing the unwanted stabilization of the open hydroxyacid form by human albumin. Moreover, a recent x-ray crystallographic analysis of the ternary complex formed by topo I, a DNA oligonucleotide and **2** showed that substituents at the 7 and 9 positions of CPT did not influence the CPT-topo I interactions [25]. The 7–10 positions can bear large groups, allowing a wide variety of structural modifications. In addition to substitution at positions 7–10, fusion of an additional ring onto the A/B ring has led to several CPT analogues in clinical trials (as depicted in Figure 2). In this study, the 10 position was chosen for substitution with either a cyclohexyl or morpholine ring (depicted in Figure 5) to investigate the effect of the water solubility of CPT on its anticancer activity. As previously discussed, the introduction of a morpholine group should increase the water solubility of CPT, and thus influence its potency and pharmacokinetic profile.

## 2. Results and Discussion

### 2.1. Synthesis of CPT Derivatives ***P210*** and ***P211***

**Scheme 1:**
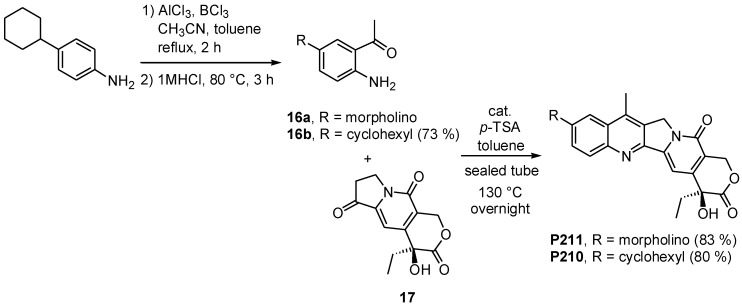
Preparation of **P210** and **P211**.

The strategy for the preparation of **P210** and **P211** is shown in Scheme 1. The condensation of commercially available 1-(2-amino-5-morpholinophenyl)-1-ethanone (**16a**) with (*S*)-4-ethyl-4-hydroxy-7-8-dihydro-1*H*-pyrano[3,4-*f*]indolizine-3,6-10(4*H*)-trione (**17**) in the presence of a catalytic amount of *p*-toluenesulfonic acid monohydrate (*p*-TSA) in toluene under reflux for 3 h produced a low yield of **P211** [26]. The *p*-TSA catalyzed condensation could be carried out in a sealed tube by heating at 130 °C overnight, affording **P211** in 83% yield. Moreover, 1-(2-amino-5-cyclohexylphenyl)ethanone (**16b**) was synthesized by the acylation of 4-cyclohexylamine [27]. Then, **P210** could be obtained at an 80% yield by the *p*-TSA catalyzed condensation of **16b** with **17** under the above-mentioned optimized conditions.

### 2.2. The Difference in Solubility between ***P210*** and ***P211***

The solubilities of **P210** and **P211** in DMSO, DMAC, 5% DMSO/95% normal saline co-solvent system, and 5% DMAC/95% normal saline co-solvent system were summarized in Table 1. 10-Cyclohexyl-7-methyl-20(*S*)-camptothecin (**P210**) exhibited higher solubilities than 7-methyl-10-morpholino-20(*S*)-camptothecin (**P211**) in polar aprotic solvents (DMSO and DMAC). However, these two camptothecin derivatives **P210** and **P211** did not exhibit apparent differences in solubility between 5% DMSO/95% normal saline co-solvent system and 5% DMAC/95% normal saline co-solvent system.

### 2.3. DFT-B3LYP Study of ***P210** (**P211**)* and Their Hydrated Complexes

**Figure 6:**
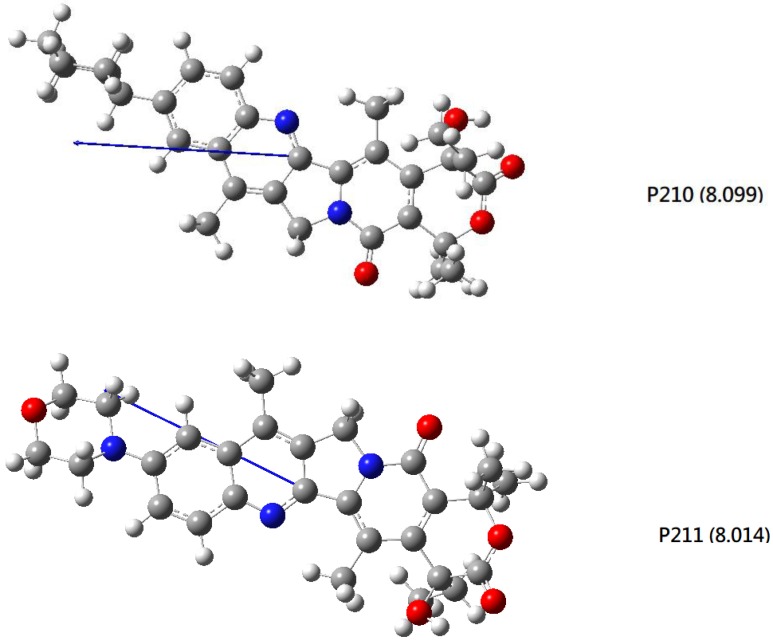
Compounds **P210** and **P211** (the values in parentheses are their respective dipole moments in Debye; carbon in gray, nitrogen in blue, oxygen in red, and hydrogen in white).

The introduction of the morpholine ring should increase the polarity of CPT, and thus, the solubility of CPT in a polar solvent, e.g. water, our solvent of interest. However, the results of the aforementioned solubility test were different. Therefore, we attempted to rationalize the differences in solubility from a theoretical aspect. We could calculate the dipole moments of both **P210** and **P211** using the B3LYP/D95V++** theoretical level to perform a gas-phase study. Generally, a more polar molecule has a larger dipole moment and better solubility in a polar solvent than a less polar molecule. The dipole moments of **P210** and **P211** are listed and compared in Figure 6, which shows that the introduction of a polar morpholine ring in position 10 of CPT slightly reduced its dipole moment and consequently its polarity.

**Figure 7:**
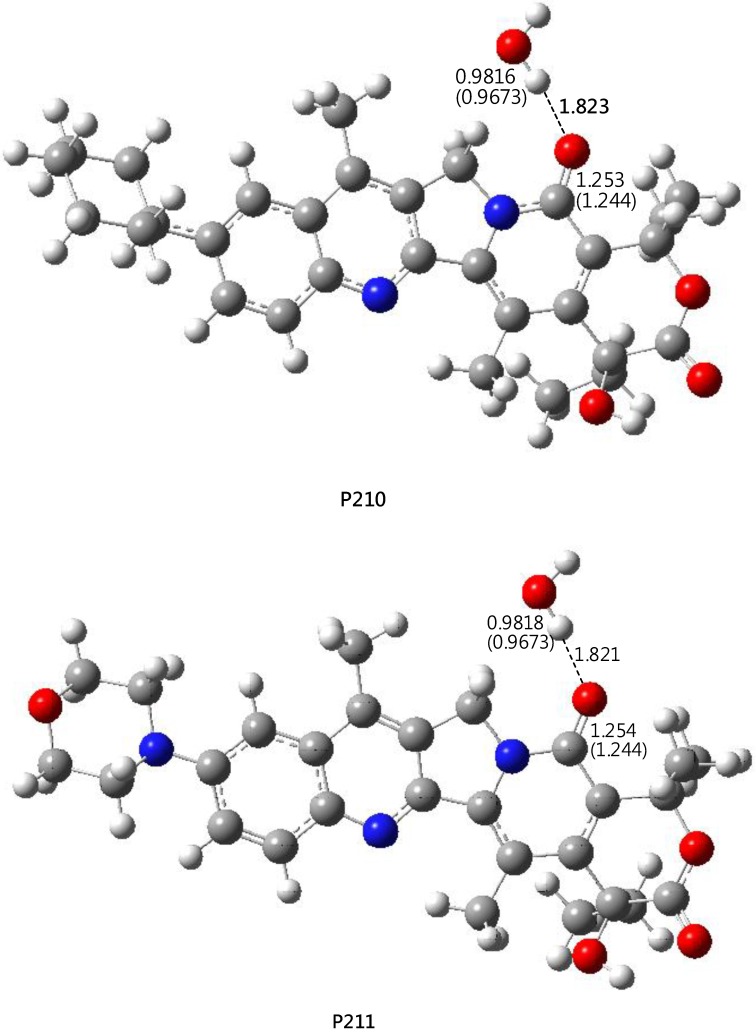

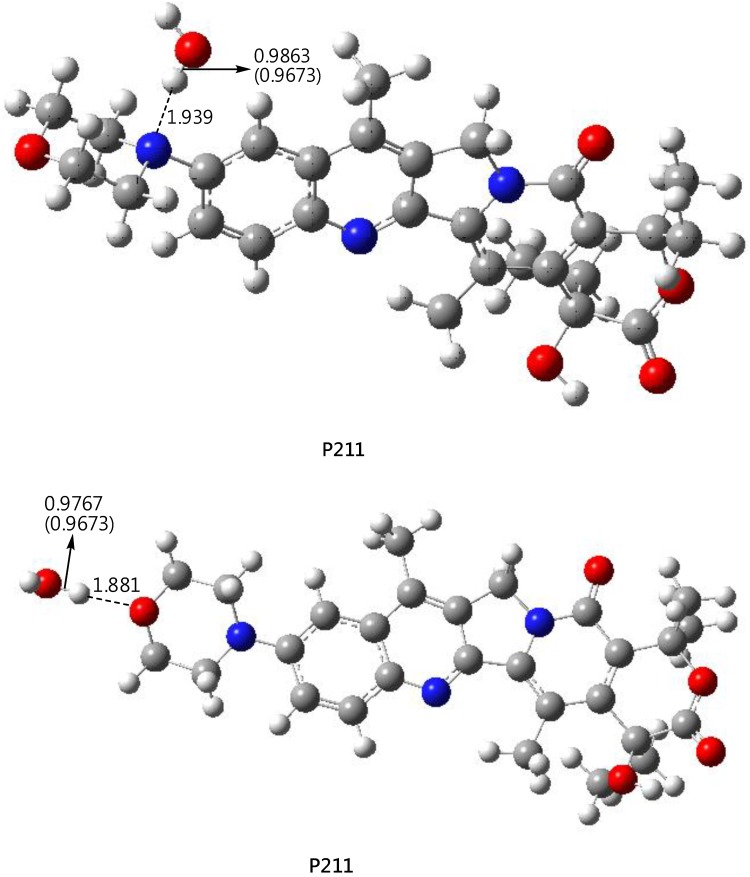
Hydrated **P210** and **P211**. Values without parentheses indicate bond lengths (Å) for the water/CPT derivative system; values in parentheses indicate bond lengths (Å) for the free molecules (carbon in gray, nitrogen in blue, oxygen in red, and hydrogen in white).

We not only compared the differences in their dipole moments, but also compared the differences in the strength of their interaction with one water molecule. A CPT derivative with stronger interaction with one water molecule should have better aqueous solubility than that with weaker interaction with one water molecule. In agreement with previous studies [28,29], both **P210** and **P211** interact most strongly with the water molecule via the carbonyl group in the D ring (Figure 7).

As depicted in Figure 7, the C=O bond length increases in both **P210** and **P211** upon interaction with a water molecule. At this theoretical level, without correcting for the basis set superposition error, the strengths of the interactions of **P210** and **P211** with a single water molecule were calculated to be −25.2 and −28.3 kJ/mol, respectively. The basis set superposition error is not corrected in this study because this correction must be practically equal for all the systems and cannot change the relative order of individual hydration energies. Apart from the carbonyl group on ring D, both **P210** and **P211** have other possible hydration sites: the oxygen atom on ring E, both C=O and OH group on ring E, and the nitrogen atom on ring B; the interaction energies of these sites for **P210** (**P211**) with a water molecule were calculated to be −13.7 (−16.8), −15.7 (−18.0), and −8.3 (−11.5) kJ/mol, respectively. Interestingly, the C=O and OH groups on ring E for both **P210** and **P211** could simultaneously form O…H hydrogen bonds with one water molecule. Such interactions were still weaker than that between the carbonyl group on ring D and a water molecule. It was worthwhile to mention that the nitrogen and oxygen atoms on the morpholine ring in **P211** can also form a hydrogen bond with one water molecule; the corresponding hydrogen bond complex is also depicted in Figure 7. Using the same theoretical method, we found that the hydrogen bond strengths with one water molecule were −4.1 and −10.9 kJ/mol for nitrogen and oxygen, respectively. This could be easily rationalized by the fact that oxygen is more electronegative than nitrogen. Although the calculated hydrogen bond lengths of **P210** and **P211** with one water molecule did not show any significant difference, **P211** could form a stronger interaction with one water molecule. Based on our recently calculated results, although a morpholine ring is a polar group, its introduction into CPT reduced the dipole moment of CPT and did not induce a large difference in the strength of the interaction between CPT and a water molecule. Therefore, **P211** might not have better aqueous solubility than **P210**.

## 3. Materials and Methods

### 3.1. General Information

Melting points (mp) were determined using a Büchi M560 apparatus (Büchi Labortechnik AG, Flawil, Switzerland) and are uncorrected. Optical rotations ([*α*]_D_) were measured using a P-2000 polarimeter (JASCO, Easton, MD, USA). Infrared (IR) spectra were reported in wave numbers (cm^−1^) using an IR Prestige-21/FTIR-8400S Fourier transform infrared spectrophotometer (Shimadzu Kyoto, Kyoto Prefecture, Japan). ^1^H and ^13^C-NMR spectra were recorded using a Bruker AVANCE III 500 FT-NMR spectrometer (Bruker, Rheinstetten, Germany) at room temperature, and the chemical shifts are reported in ppm using tetramethylsilane (TMS) as the internal standard. Mass spectra (MS) were obtained using a LCQ ion-trap mass spectrometer using the electrospray ionization (ESI) technique (Thermo-Finnigan, Mundelein, IL, USA). High-resolution electrospray ionization mass spectra (HRESI-MS) were recorded on a LTQ orbitrap XL hybrid ion trap-orbitrap mass spectrometer (Thermo Scientific, Waltham, MA, USA). Column chromatography was performed on 230−400 mesh silica gel.

### 3.2. Synthesis of 1-(2-Amino-5-cyclohexylphenyl)ethanone (8)

To a slurried mixture of 4-cyclohexylaniline (175.3 mg, 1.0 mmol) and anhydrous aluminum chloride (186.7 mg, 1.4 mmol) in dry toluene (6 mL) was added boron trichloride (1 M in hexane, 6.9 mL, 1.2 mmol) at room temperature under N_2_. Acetonitrile (2.6 mL, 50 mmol) was added to the reaction mixture, and the mixture was refluxed under N_2_ for 3 h. After cooling, 1M aqueous hydrochloric acid (2 mL) was added to the resulting mixture at room temperature. The mixture was stirred at 80 °C for 0.5 h, and then poured into ice water. A 5 M aqueous sodium hydroxide solution was added to increase the pH of the resultant solution to >13, and the mixture was extracted with EtOAc (30 mL × 3). The combined organic layers were washed with brine (10 mL × 1), dried over MgSO_4_, filtered, and concentrated. The crude product was purified by column chromatography using silica gel and hexane−EtOAc (20:1 *v/v*) as the eluent, affording the pure product 8 as an off-white solid (158.6 mg, 73% yield). mp 69–69.5 °C; IR (KBr) ν_max_ 3429, 3329, 2924, 2846, 1643, 1632, 1547 cm^−1^; ^1^H-NMR (500 MHz, CDCl_3_) δ 1.17–1.44 (5H, m), 1.70–1.90 (5H, m), 2.35–2.45 (1H, m), 2.60 (3H, s), 6.12 (2H, br s), 6.59 (1H, d, *J* = 8.4 Hz), 7.14 (1H, d, *J* = 8.4 Hz), 7.50 (1H, s); ^13^C-NMR (125 MHz, CDCl_3_) δ 26.0, 26.8 (C × 2), 27.8, 34.6 (C × 2), 43.5, 117.2, 118.1, 129.4, 133.3, 135.4, 148.4, 200.7; ESIMS *m/z* (rel. int.) 218 (100, [M + H]^+^); HRESIMS *m/z* calcd for C_14_H_20_NO 218.1539; found 218.1541 [M + H]^+^.

### 3.3. General Procedure for the Condensation of 16 with 17

A mixture of compound **16a** or **16b** (0.1 mmol), (*S*)-4-ethyl-4-hydroxy-7-8-dihydro-1*H*-pyrano-[3,4-*f*]indolizine-3,6-10(4*H*)-trione (**17**, 0.2 mmol), and *p*-TSA (0.02 mmol) in dry toluene (6 mL) was placed in a sealed tube and then heated at 130 °C overnight. After cooling, the resulting solution was directly purified by column chromatography using silica gel and pure CHCl_3_ or CH_2_Cl_2_-MeOH (40:1 *v/v*) as the eluent, affording the pure camptothecin derivative **P210** or **P211**.

*10-Cyclohexyl-7-methyl-20(S)-camptothecin* (**P210**). Eluted with pure CHCl_3_; yield 80%; pale yellow solid; mp 243–245 °C; [*α*]_D_ +37.3° (c 0.32, CHCl_3_); IR (KBr) ν_max_ 3433, 3337, 2924, 2851, 1748, 1659, 1597 cm^−1^; ^1^H-NMR (500 MHz, CDCl_3_) δ 1.03 (3H, t, *J* = 7.4 Hz), 1.29–1.38 (1H, m), 1.42–1.62 (4H, m), 1.80–1.94 (5H, m), 1.99–2.02 (2H, m), 2.73–2.80 (1H, m), 2.76 (3H, s), 3.98 (1H, s), 5.21 (2H, s), 5.29 (1H, d, *J* = 16.2 Hz), 5.73 (1H, d, *J* = 16.2 Hz), 7.65 (1H, s), 7.69 (1H, d, *J* = 8.7 Hz), 7.82 (1H, s), 8.14 (1H, d, *J* = 8.7 Hz); ^13^C-NMR (125 MHz, CDCl_3_) δ 7.8, 15.3, 26.1, 26.8 (C × 2), 31.7, 34.4 (C × 2), 45.0, 49.8, 66.4, 72.8, 97.8, 118.2, 120.2, 127.6, 128.0, 130.2, 130.3, 139.1, 147.3, 147.8, 147.9, 150.2, 150.8, 157.7, 173.9; ESIMS *m/z* (rel. int.) 445 (100, [M + H]^+^), 401 (24), 381 (30); HRESIMS *m/z* calcd for C_27_H_29_N_2_O_4_ 445.2122; found 445.2123 [M + H]^+^.

*7-Methyl-10-morpholino-20(S)-camptothecin* (**P211**). Eluted with CH_2_Cl_2_-MeOH (40:1 *v/v*); yield 83%; yellow solid; mp 283–285 °C; [*α*]_D_ +11.3° (c 0.34, CHCl_3_); IR (KBr) ν_max_ 3414, 1744, 1655, 1593 cm^−1^; ^1^H-NMR (500 MHz, CDCl_3_) δ 1.03 (3H, t, *J* = 7.2 Hz), 1.80–1.98 (2H, m), 2.70 (3H, s), 3.38 (4H, t, *J* = 4.8 Hz), 3.85 (1H, s), 3.95 (4H, t, *J* = 4.8 Hz), 5.19 (2H, s), 5.30 (1H, d, *J* = 16.1 Hz), 5.74 (1H, d, *J* = 16.1 Hz), 7.14 (1H, s), 7.55 (1H, d, *J* = 9.3 Hz), 7.58 (1H, s), 8.10 (1H, d, *J* = 9.3 Hz); ^13^C-NMR (125 MHz, CDCl3) δ 7.8, 15.4, 31.6, 48.8 (C × 2), 49.8, 66.4, 66.7 (C × 2), 72.9, 97.2, 104.2, 117.5, 122.0, 128.2, 129.3, 131.3, 137.2, 144.3, 147.5, 148.8, 150.1, 150.2, 157.7, 174.0; ESIMS *m/z* (rel. int.) 448 (100, [M + H]^+^), 404 (13), 381 (79); HRESIMS *m/z* calcd for C_25_H_26_N_3_O_5_ 448.1867; found 448.1871 [M + H]^+^.

### 3.4. The Solubility Test

To test the difference in solubility between **P210** and **P211**, four solvents were chosen in this study for a systematic comparison; namely, DMSO, DMAC, 5% DMSO/95% normal saline (*v/v*), and 5% DMAC/95% normal saline (*v/v*). 

### 3.5. DFT-B3LYP Study of P210 (P211) and Their Hydrated Complexes

All DFT calculations were carried out by the program Gaussian 16 (Wallingford, CT, USA) [30]. The DFT calculation was performed by the hybrid B3LYP method, which is based on the method proposed by Becke and a mixture of the exact (Hartree-Fock, HF) and DFT exchange utilizing the B3 functional was considered, together with the LYP correlation functional [31,32]. The B3LYP calculations were carried out using the basis set D95V++** [33]. After obtaining the converged geometry, the harmonic vibrational frequencies were calculated the same theoretical level to confirm whether the number of imaginary frequencies is zero for the stationary points.

## 4. Conclusions

Since water solubility is an important factor in drug development, we synthesized two CPT derivatives, **P210** and **P211**, and compared their solubilities. To rationalize the differences in their solubilities, we subjected them and their hydrated complexes to DFT-B3LYP comparison. Based on our recent computational results, the carbonyl group on ring D (Figure 1) in both **P210** and **P211** formed the strongest interactions with a water molecule. Although a morpholine ring is a polar group, its introduction into CPT reduced the dipole moment of CPT and did not induce large differences in the interaction strength between CPT and a water molecule. Therefore, **P211** might not be more soluble in water than **P210**. In-vivo and in-vitro studies on the differences in their anticancer activities are in progress.

## Figures and Tables

**Table 1:** The solubilities of **P210** and **P211** (in mg/1 mL).

	P210	P211
DMSO	10.6 sparingly soluble	7.2 slightly soluble
DMAC	49.5 soluble	13.2 sparingly soluble
5% DMSO + 95% normal saline	0.10 very slightly soluble	0.10 very slightly soluble
5% DMAC + 95% normal saline	0.10 very slightly soluble	0.10 very slightly soluble
